# Progress Toward Poliomyelitis Eradication ― Afghanistan, January 2020–November 2021

**DOI:** 10.15585/mmwr.mm7103a3

**Published:** 2022-01-21

**Authors:** Katrin S. Sadigh, Irfan Elahi Akbar, Mufti Zubair Wadood, Hemant Shukla, Jaume Jorba, Sumangala Chaudhury, Maureen Martinez

**Affiliations:** ^1^Epidemic Intelligence Service, CDC; ^2^Global Immunization Division, Center for Global Health, CDC; ^3^Polio Eradication Department, World Health Organization, Kabul, Afghanistan; ^4^Polio Eradication Department, World Health Organization, Geneva, Switzerland; ^5^Polio Eradication Department, World Health Organization, Amman, Jordan; ^6^Division of Viral Diseases, National Center for Immunization and Respiratory Diseases, CDC.

Wild poliovirus types 2 and 3 were declared eradicated in 2015 and 2019, respectively, and, since 2017, transmission of wild poliovirus type 1 (WPV1) has been detected only in Afghanistan and Pakistan. In 2020, these countries reported their highest number of WPV1 cases since 2014 and experienced outbreaks of type 2 circulating vaccine-derived poliovirus (cVDPV2)* (*1*); in Afghanistan, the number of WPV1 cases reported increased 93%, from 29 in 2019 to 56 in 2020, with 308 cVDPV2 cases reported. This report describes the activities and progress toward polio eradication in Afghanistan during January 2020–November 2021 and updates previous reports (*2*–*4*). Despite restrictions imposed by antigovernment elements since 2018, disruption of polio eradication efforts by the COVID-19 pandemic, and civil and political instability, eradication activities have resumed. During January–November 2021, four WPV1 cases and 43 cVDPV2 cases were detected, representing decreases of 93% from 56 and 85% from 281, respectively, during the same period in 2020. After the assumption of nationwide control by the current de facto government of Afghanistan during August 2021, health officials committed to oral poliovirus vaccine (OPV) campaigns nationwide, with the potential to vaccinate approximately 2.5 million children against poliovirus who were previously not accessible for ≥2 years. Although challenges remain, vigorous, sustained polio eradication efforts in Afghanistan could result in substantial progress toward eradication during 2022–2023.

## Immunization Activities

The estimated national routine vaccination coverage with the third dose of bivalent OPV (bOPV containing Sabin types 1 and 3) (OPV3) among children aged 12 months was 73% during 2018 and 2019; the estimated 1-dose coverage with injectable inactivated poliovirus vaccine (IPV) during 2019 was 66% (*5*). Nationwide, during 2020 and 2021 to date, 27% of children aged 6–59 months with nonpolio acute flaccid paralysis (NPAFP; paralysis with no evidence of poliovirus infection, a proxy indicator of OPV3 coverage) had received 3 OPV doses through routine immunization services, based on caretaker recall. The percentage of children aged 6–59 months with NPAFP who never received OPV through routine or supplementary immunization activities (SIAs)^†^ increased from 1% in 2019 to 4% in 2020, and to 6% in 2021, with the highest provisional percentages in 2021 in the southern provinces of Zabul (32%), Nimroz (13%), and Helmand (21%), and the western province of Badghis (19%). However, this proportion remained at or near 0% in most of the eastern provinces during 2019–2021 and decreased from 10% and 4.9% in the southeast provinces of Paktya and Khost to 0% and 2.4%, respectively.

During January 2020–November 2021, 10 OPV SIAs were conducted; eight were national immunization days (NIDs) and two were subnational immunization days (SNIDs) targeting children aged <5 years. In addition, four case-response campaigns with type 1-containing monovalent OPV (mOPV), bOPV, or trivalent OPV (tOPV containing Sabin types 1, 2, and 3) were implemented during July–November 2020. During January and February 2020, during IPV fixed-site campaigns, IPV was administered to 159,833 (93%) children targeted in the accessible districts in the eastern provinces of Kunar, Nangarhar, and Laghman, and the southeast province of Paktika.

Most districts of the southern and eastern provinces of Afghanistan were under control of antigovernment elements before assumption of full nationwide control by the de facto government of Afghanistan during August 2021. Children who are unvaccinated are classified as being inaccessible to vaccination or as accessible but missed.^§^ House-to-house SIAs, the optimal method for reaching every child for OPV vaccination, have been banned in all areas controlled by antigovernment elements since May 2018. Enhanced transit point and fixed-post vaccination at health facilities have been permitted since October 2019.

According to administrative data, an estimated 2,752,578 (28%) of the 9,999,227 children aged <5 years were inaccessible to vaccination during the January 2020 NID. In October 2020, when SIAs recommenced after a 5-month suspension because of COVID-19, this number increased to 3,381,642 (34%) and peaked at approximately 4,000,000 (40%) during the March and June 2021 NIDs. During these SIAs, the proportion of children reported as accessible but missed ranged from 4% in February 2020 to 3% in October 2020.

Lot quality assurance sampling (LQAS)^¶^ surveys assess SIA quality in accessible areas. On the basis of the number of unvaccinated children among those surveyed, SIAs in districts either passed (90%) or failed. The proportion of surveyed districts with failed SIAs during January 2020–June 2021 ranged from 40% in July 2020 to 12% in November 2020, January 2021, and June 2021.

Children aged ≤10 years are also targeted for vaccination along major travel routes throughout Afghanistan, and persons of all ages are targeted at border crossing points with Iran and Pakistan. During January 2020–November 2021, 14,899,633 doses of bOPV were administered to children at transit points and 1,432,964 doses to persons of all ages at border crossings.

## Poliovirus Surveillance

**Acute flaccid paralysis surveillance.** Detection of two or more NPAFP cases per 100,000 persons aged <15 years together with ≥80% of AFP cases having adequate stool specimens collected** indicate that surveillance is sufficiently sensitive to detect poliovirus cases. The Afghanistan AFP surveillance network includes 2,843 health facilities and 45,029 community- and health facility–based reporting volunteers. During 2020, the national NPAFP rate was 22 per 100,000 persons aged <15 years in accessible areas and 20 per 100,000 in inaccessible areas (regional range = 12–24) (Table). The percentages of AFP cases with adequate specimens were 95% and 92% in accessible and inaccessible areas, respectively (regional range = 86%–98%).

**TABLE:** Acute flaccid paralysis surveillance performance indicators, reported cases of wild poliovirus and vaccine-derived poliovirus type 2,* and percentage of environmental samples with detection of wild poliovirus type 1, by region and period ― Afghanistan, January 2020–November 2021^†^

Region	AFP surveillance indicators						No. of WPV1 cases reported			No. of cVDPV2 cases reported			No. (%) of ES samples with WPV1 detected^§^		
	No. of AFP cases		NPAFP rate (%)^¶^		% with adequate stool specimens**		2020		2021	2020		2021	2020		2021
	2020	2021	2020	2021	2020	2021	Jan–Jun	Jul–Dec	Jan–Nov	Jan–Jun	Jul–Dec	Jan–Nov	Jan–Jun	Jul–Dec	Jan–Nov
**All regions**	**3,972**	**3,009**	**18**	**17**	**93**	**94**	**34**	**22**	**4**	**54**	**254**	**43**	**22 (11)**	**13 (6)**	**1 (0.3)**
Badakhshan	83	61	12	10	89	93	1	0	0	1	0	0	0 (―)	0(―)	0 (―)
Central	734	658	15	17	98	98	0	0	0	0	17	4	0 (―)	1 (3)	0 (―)
Eastern	543	374	24	20	92	96	2	0	0	51	19	0	2 (2)	2 (3)	0 (―)
Northeastern	429	298	18	15	95	94	0	0	3	0	4	0	0 (―)	1 (20)	0 (―)
Northern	337	255	13	12	91	89	1	0	0	0	7	2	0 (―)	0 (―)	0 (―)
Southeastern	426	283	20	15	95	96	0	6	1	1	33	8	1 (8)	0 (―)	0 (―)
Southern	798	586	21	18	86	89	23	15	0	0	145	12	17 (26)	7 (9)	1 (0.8)
Western	622	494	20	20	93	93	7	1	0	1	29	17	2 (29)	2 (40)	0 (―)

**Abbreviations:** AFP = acute flaccid paralysis; cVDPV2 = circulating vaccine-derived poliovirus type 2; ES = environmental surveillance; NPAFP = nonpolio acute flaccid paralysis; WPV1 = wild poliovirus type 1.

* cVDPVs are genetically linked VDPV2 isolates for which there is evidence of person-to-person transmission within the community.

^†^ Data as of January 11, 2022.

^§^ Total number of ES samples by period: January 2020–June 2020 = 208, July 2020–December 2020 = 205, and January 2021–November 2021 = 341. WPV-1–positive ES samples were detected in 2020 in Kabul (central), Nangarhar (eastern), Kunduz (northeastern), Khost (southeastern), and Helmand and Kandahar (southern) provinces, and in 2021 in Helmand (southern) province. Percentages indicate specimens testing positive for WPV1 for the total number of specimens collected for all regions and the specific region during that period.

^¶^ Cases per 100,000 persons aged <15 years. The surveillance performance indicator target is ≥2 NPAFP cases per 100,000 persons aged <15 years.

** Surveillance performance indicator target is ≥80% of AFP cases have adequate stool specimens collected. Adequate stool specimens are defined as two stool specimens of sufficient quality for laboratory analysis, collected ≥24 hours apart, both within 14 days of paralysis onset, and arriving in good condition at a World Health Organization-accredited laboratory with reverse cold chain maintained, without leakage or desiccation, and with proper documentation.

**Environmental surveillance.** Poliovirus surveillance in Afghanistan is supplemented by environmental surveillance (ES) conducted through the systematic sampling and virologic testing of sewage at 25 sites in 13 provinces. During 2019, WPV1 was detected in ES specimens from sites in Helmand and Kandahar in the southern region and Nangarhar in the eastern region. During 2020, detection of WPV1-postitive ES specimens expanded in geographic scope to include Khost in the southeastern, Kabul in the central, Herat in the western, and Kunduz in the northeastern regions. One WPV1 ES-positive sample was detected during January–November 2021, a 97% decrease compared with 34 detected during the same period in 2020. Regarding cVDPV2^††^ isolations, ES specimens in 2020 tested positive from sites in 10 provinces: Helmand and Kandahar southern provinces; Nangarhar, Kunar, and Laghman eastern provinces; Khost and Paktika southeastern provinces; and Kabul in central, Herat in western, and Kunduz in northwestern provinces. During 2021, cVDPV2-positive ES specimens were detected in only six provinces: Helmand, Kandahar, Nangarhar, Kabul, Herat, and Kunduz.

## Epidemiology of Poliovirus Cases and Genomic Sequence Analysis of Poliovirus Isolates

**Figure 1:**
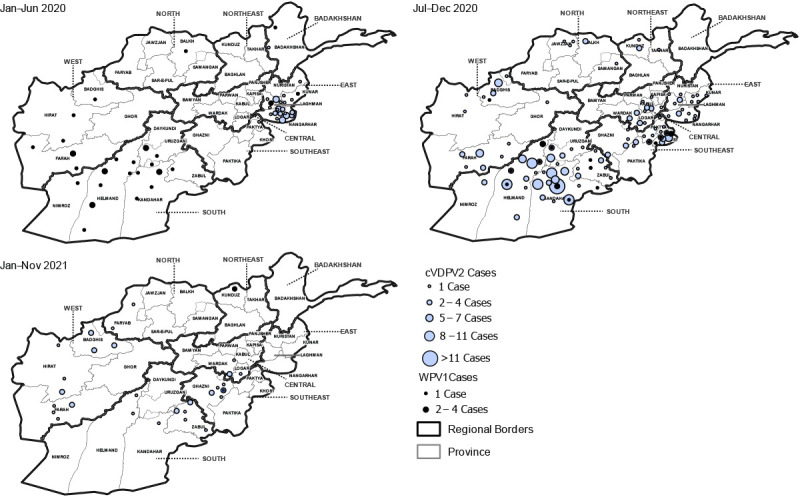
Cases of wild poliovirus type 1 and circulating vaccine-derived poliovirus type 2,*^,†^ by province and period — Afghanistan, January 2020–November 2021^§^ **Abbreviations:** cVDPV2 = circulating vaccine-derived poliovirus type 2; WPV1 = wild poliovirus type 1. * cVDPVs are genetically linked VDPV2 isolates for which there is evidence of person-to-person transmission in the community. ^†^ Total cases by period: January–June 2020 = 34 WPV1 and 54 cVDPV2; July–December 2020 = 22 WPV1 and 254 cVDPV2; and January–November 2021 = 4 WPV1 and 43 cVDPV2. ^§^ Data as of January 11, 2022.

**Figure 2:**
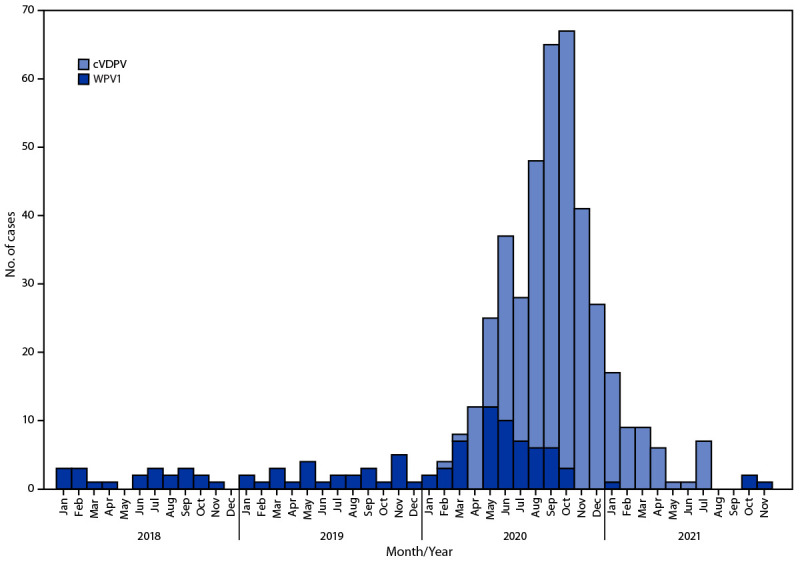
Number of wild poliovirus type 1 cases (n = 60) and circulating vaccine-derived poliovirus type 2* cases, by month of onset of paralysis (n = 351) — Afghanistan, January 2020–November 2021^†^ **Abbreviations:** cVDPV2 = circulating vaccine-derived poliovirus type 2; WPV1 = wild poliovirus type 1. * cVDPVs are genetically linked VDPV2 isolates for which there is evidence of person-to-person transmission in the community. ^†^ Data as of January 11, 2022.

During 2020, WPV1 cases increased in number and geographic distribution compared with 2019: 56 WPV1 cases were reported from 38 districts in 14 provinces in 2020, compared with 29 WPV1 cases reported from 20 districts in 10 provinces in 2019. During January–November 2021 (as of January 11, 2022), only four WPV1 cases were reported (Table) (Figure 1) (Figure 2). Twenty-one (35%) of 60 patients with WPV1 cases reported between January 2020 and November 2021 had never received OPV, 14 (23%) had received 1 or 2 doses, and 24 (40%) had received ≥3 doses; 23 (38%) had never received OPV through routine immunization but had received ≥1 SIA doses.

Genomic sequence analysis of the VP1 capsid protein of poliovirus isolates provided evidence for multiple episodes of cross-border transmission between Afghanistan and Pakistan during 2018–2021, with sustained local transmission in both countries. During January 2020–November 2021, nine (15%) of 60 WPV1 isolates from AFP patients and nine (25%) of 36 WPV1 ES isolates in Afghanistan had the closest genetic links to WPV1 isolates from Pakistan; the remaining were most closely linked to AFP and ES isolates from within Afghanistan (Table). During the same period, five WPV1 genetic clusters (groups of viruses sharing ≥95% VP1 sequence identity) were detected among AFP cases. Although transmission in the eastern and southern provinces is mostly from distinct genetic clusters, two WPV1 isolates were identified in the south from clusters originally identified in the east. Sixteen orphan WPV1 viruses^§§^ were isolated from ES or AFP cases, signaling gaps in AFP surveillance during this period, but similar in percentage to the report for the overlapping period of January 2019–July 2020 (*2*).

During January 2020–November 2021, with importation of cVDPV2 from Pakistan and new emergences seeded after mOPV2 use in Afghanistan (*6*), 351 cVDPV2 cases were reported from 131 districts in 28 provinces; 225 (64%) of those occurred among children aged <36 months. Of the 351 cVDPV2 cases, 225 (64%) were genetically related to the PAK-GB-1 emergence first detected in Gilgit-Baltistan, Pakistan, 127 (36%) were related to the AFG-NGR-1 emergence first detected in Afghanistan’s Nangarhar province, and four (1%) were related to the cVDPV2 AFG-HLD-1 emergence first detected in Helmand province (*7*).

## Discussion

Afghanistan and Pakistan remain the only countries with endemic WPV1 transmission; substantive progress in these countries represents progress toward global polio eradication. Although the overall number of WPV1 cases in Afghanistan was high in 2020, there was a marked decrease in cases from the first to the second half of the year and case numbers declined further during 2021. Although the number of inaccessible, and therefore unvaccinated, children markedly increased in 2021, WPV1 transmission decreased, possibly because of decreased population mixing and movement during the early phases of the COVID-19 pandemic and rapid return to quality SIAs.

The findings in this report are subject to at least two limitations. First, for the November 2021 SIA, the accuracy of the reported coverage data and LQAS surveys data is uncertain because many of these are reported by inexperienced officers selected by the de facto government without other oversight. Second, the quality of AFP surveillance likely suffered since the beginning of the COVID-19 pandemic and might also remain reduced from potential disruptions since the transition in government; however, a decrease in the proportion of WPV1-positive ES isolates in 2021 to date suggests that the current AFP surveillance data are consistent with decreased transmission.

In addition to the four WPV1 cases reported from Afghanistan during 2021, as of January 11, 2022, only one WPV1 case has been reported from Pakistan, further evidence for decreased transmission within the shared epidemiologic block. Because the de facto government of Afghanistan has resumed intensive OPV vaccination, the number of inaccessible children should be greatly decreased. House-to-house polio vaccination resumed in portions of the country with the involvement of female frontline workers in November 2021, and a second campaign took place in December 2021 synchronized with Pakistan. If future efforts are robust, sustained, and implemented countrywide, substantial progress toward interrupting WPV1 transmission in Afghanistan is possible during 2022–2023.

**Summary**

**What is already known about this topic?**

Wild poliovirus circulation continues only in Afghanistan and Pakistan.

**What is added by this report?**

Despite an increase in the numbers of inaccessible children in Afghanistan in 2021 and disruption of polio eradication activities caused by the COVID-19 pandemic and abrupt changes in government, the number of wild poliovirus type 1 cases and percentage of positive sewage samples have markedly decreased by 93% and 97%, respectively, from the same period in 2020.

**What are the implications for public health practice?**

Although challenges remain, prospects for vaccination of previously inaccessible children along with sustained, robust polio eradication efforts in Afghanistan could result in substantial progress toward eradication during 2022–2023.

## Footnotes

* cVDPV can emerge when attenuated OPV virus reverts to neurovirulence as a result of transmission in areas with low immunization coverage.

† SIAs are mass house-to-house campaigns targeting children aged <5 years with OPV, regardless of their vaccination history.

§ Children living in antigovernment element–held areas with insecurity or where SIAs were banned up to this time are classified as being inaccessible to vaccination. Children in areas that were fully accessible for OPV SIAs were classified as having been missed if they remained unvaccinated because of absence from home, refusal, or low-quality campaign implementation by vaccination teams.

¶ LQAS is a rapid survey method to assess the quality of vaccination activities after SIAs in predefined areas, such as health districts (referred to as “lots”), using a sample size of 60. LQAS involves dividing the population into lots and ascertaining receipt of vaccination by randomly selecting children within each lot. If the number of unvaccinated persons in the sample exceeds three, then the SIA quality in that area is classified as failed (i.e., at a pass threshold of ≥90%) and mop-up activities are recommended. If the threshold of ≥90% is met, the SIA’s quality for the area is classified as having passed, although mop-up activities might still be indicated in certain areas.

** The global standard surveillance performance indicator target is ≥80% of AFP cases with adequate stool specimens collected. Adequate stool specimens are defined as two stool specimens of sufficient quality for laboratory analysis, collected ≥24 hours apart, both within 14 days of paralysis onset, and arriving in good condition at a World Health Organization-accredited laboratory with reverse cold chain maintained, without leakage or desiccation, and with proper documentation.

†† cVDPV2s are genetically linked VDPV2 isolates for which there is evidence of person-to-person transmission in the community.

§§ Orphan viruses are ≥1.5% divergent from their closest genetic match (i.e., ≤98.5% identity).

## References

[1] Chard AN, Datta SD, Tallis G, Progress toward polio eradication—worldwide, January 2018–March 2020. MMWR Morb Mortal Wkly Rep 2020;69:784–9. 10.15585/mmwr.mm6925a4 32584798 PMC7316320

[2] Martinez M, Akbar IE, Wadood MZ, Shukla H, Jorba J, Ehrhardt D. Progress toward poliomyelitis eradication—Afghanistan, January 2019–July 2020. MMWR Morb Mortal Wkly Rep 2020;69:1464–8. 10.15585/mmwr.mm6632a5 33031360 PMC7561224

[3] Martinez M, Shukla H, Nikulin J, Mbaeyi C, Jorba J, Ehrhardt D. Progress toward poliomyelitis eradication—Afghanistan, January 2018–May 2019. MMWR Morb Mortal Wkly Rep 2019;68:729–33. 10.15585/mmwr.mm6632a5 31437144 PMC6705892

[4] Martinez M, Shukla H, Nikulin J, Progress toward poliomyelitis eradication—Afghanistan, January 2016–June 2017. MMWR Morb Mortal Wkly Rep 2017;66:854–8. 10.15585/mmwr.mm6632a5 28817551 PMC5657670

[5] World Health Organization. WHO vaccine-preventable diseases: monitoring system. 2020 global summary. Geneva, Switzerland: World Health Organization; 2020. https://apps.who.int/immunization_monitoring/globalsummary/countries?countrycriteria%5Bcountry%5D%5B%5D=AFG

[6] Alleman MM, Jorba J, Henderson E, Update on vaccine-derived poliovirus outbreaks—worldwide, January 2020–June 2021. MMWR Morb Mortal Wkly Rep 2021;70:1691–9. 10.15585/mmwr.mm7049a1 34882653 PMC8659190

[7] Hsu CH, Rehman MS, Bullard K, Progress toward poliomyelitis eradication—Pakistan, January 2019–September 2020. MMWR Morb Mortal Wkly Rep 2020;69:1748–52. 10.15585/mmwr.mm6946a5 33211676 PMC7676637

